# Hsp60 Is Actively Secreted by Human Tumor Cells

**DOI:** 10.1371/journal.pone.0009247

**Published:** 2010-02-16

**Authors:** Anna M. Merendino, Fabio Bucchieri, Claudia Campanella, Vito Marcianò, Anna Ribbene, Sabrina David, Giovanni Zummo, Giosalba Burgio, Davide F. V. Corona, Everly Conway de Macario, Alberto J. L. Macario, Francesco Cappello

**Affiliations:** 1 Dipartimento di Biomedicina Sperimentale e Neuroscienze Cliniche, Università degli Studi di Palermo, Palermo, Italy; 2 Dipartimento di Biologia Cellulare e dello Sviluppo, Università degli Studi di Palermo, Palermo, Italy; 3 Dulbecco Telethon Institute, Palermo, Italy; 4 Center of Marine Biotechnology, University of Maryland Biotechnology Institute, Baltimore, Maryland, United States of America; 5 Istituto EuroMEditerraneo di Scienza e Tecnologia, Palermo, Italy; Institut Pasteur, France

## Abstract

**Background:**

Hsp60, a Group I mitochondrial chaperonin, is classically considered an intracellular chaperone with residence in the mitochondria; nonetheless, in the last few years it has been found extracellularly as well as in the cell membrane. Important questions remain pertaining to extracellular Hsp60 such as how generalized is its occurrence outside cells, what are its extracellular functions and the translocation mechanisms that transport the chaperone outside of the cell. These questions are particularly relevant for cancer biology since it is believed that extracellular chaperones, like Hsp70, may play an active role in tumor growth and dissemination.

**Methodology/Principal Findings:**

Since cancer cells may undergo necrosis and apoptosis, it could be possible that extracellular Hsps are chiefly the result of cell destruction but not the product of an active, physiological process. In this work, we studied three tumor cells lines and found that they all release Hsp60 into the culture media by an active mechanism independently of cell death. Biochemical analyses of one of the cell lines revealed that Hsp60 secretion was significantly reduced, by inhibitors of exosomes and lipid rafts.

**Conclusions/Significance:**

Our data suggest that Hsp60 release is the result of an active secretion mechanism and, since extracellular release of the chaperone was demonstrated in all tumor cell lines investigated, our observations most likely reflect a general physiological phenomenon, occurring in many tumors.

## Introduction

Human Hsp60, the product of the *HSPD1* gene, is a Group I mitochondrial chaperonin, phylogenetically related to bacterial GroEL. Recently, the presence of Hsp60 outside the mitochondria and outside the cell, e.g. in circulating blood, has been reported [1], [2]. Although it is assumed that Hsp60 extra-mitochondrial molecule is identical to the mitochondrial one, this has not yet been fully elucidated. Despite the increasing amount of experimental evidences showing Hsp60 outside the cell, it is not yet clear how general this process is and what are the mechanisms responsible for Hsp60 translocation outside the cell. Neither of these questions has been definitively answered, whereas there is some information regarding extracellular Hsp70. This chaperone was also classically regarded as an intracellular protein like Hsp60, but in the last few years considerable evidences showed its pericellular and extracellular residence [3], [4]. Furthermore, it has been reported that extracellular Hsp70 has a role in regulating certain aspects of the immune response and in tumor growth and dissemination [3]. Unfortunately, information on secretion of Hsp60 by tumors is scarce in what concerns frequency of the phenomenon, mechanism, and physiopathological role. Here, we report results of experiments aimed at determining whether tumor cells secrete Hsp60 and whether this is an active physiological mechanism. Our data strongly suggest that extracellular Hsp60 release is the result of an active secretion mechanism not due to cell damage or death with membrane disruption, probably reflecting a general physiological phenomenon.

## Results and Discussion

### Tumor Cells Release Hsp60 and Hsp70 into the Extracellular Culture Medium

Hsp60 and Hsp70 were detected in all samples, including specific immunoprecipitates and exosomes purified from culture media, and whole-cell lysates, obtained from the tumor cell lines H292, A549 and K562 (see Materials and Methods).

**Figure 1:**
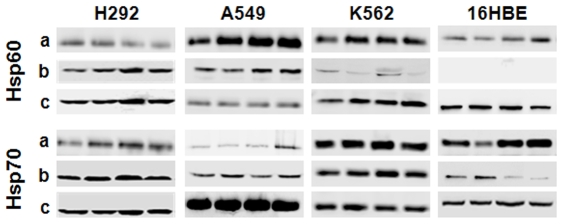
Extracellular release of Hsp60 and Hsp70 by tumor cells. The two Hsps were found extracellularly in specific immunoprecipitates from conditioned media (**a**) and in exosomes purified from the conditioned media from all three tumor cells tested (in A549, Hsp70 levels in immunoprecipitates from conditioned media were lower than in the other cell lines) (**b**). Likewise, Hsp60 and Hsp70 were present in immunoprecipitates from conditioned medium from the 16HBE non-tumor cells. However, while Hsp70 was present in the exosomes purified from the 16HBE conditioned medium, Hsp60 was not. As expected, the two Hsps were present intracellularly in all cell lines, as shown by the results with whole-cell lysates (**c**). Each set of four Western Blot lanes represents four separate experiments. In conclusion, the two Hsps were present intracellularly and were released into the extracellular space by the tumor and non-tumor cells, but Hsp60 was not secreted via exosomes by the non-tumor cells in contrast to Hsp70.

Similar results were obtained with the non-tumor 16HBE cell line with the exception of exosomes, which did not show detectable levels of Hsp60 (Figure 1). Hsp70 was present in all tested exosomal samples from tumor and non-tumor cell lines, confirming previous results and reaffirming the notion that this Hsp is a reliable marker of exosomes [5]. The presence and quality of exosomes in our preparations was further verified by transmission electron microscopy (TEM), and by determining acetylcholinesterase (AChE) activity and expression of Alix protein.

A noteworthy finding from these experiments was that Hsp60 was detected in exosomes from all the tumor cell lines tested but not in the exosomes of the non-tumor 16HBE cells, suggesting that spontaneous release of this molecule usually occurs in tumor cells, possibly reflecting their higher intracellular levels of Hsp60, which might be due to overexpression of the *hsp60* (*HSPD1*) gene and/or other mechanisms such as increased translation rate and/or increased mRNA or protein stability.

### Exosome Characterization

The results shown in Figure 1 indicate that Hsp60 and Hsp70 were found in extracellular exosomes. To further ascertain the identity of the exosomes obtained from the tumor and non-tumor cells, exosomal preparations were examined by TEM and tested for markers considered typical of exosomes.

#### a) Transmission electron microscopy of purified exosomes

**Figure 2:**
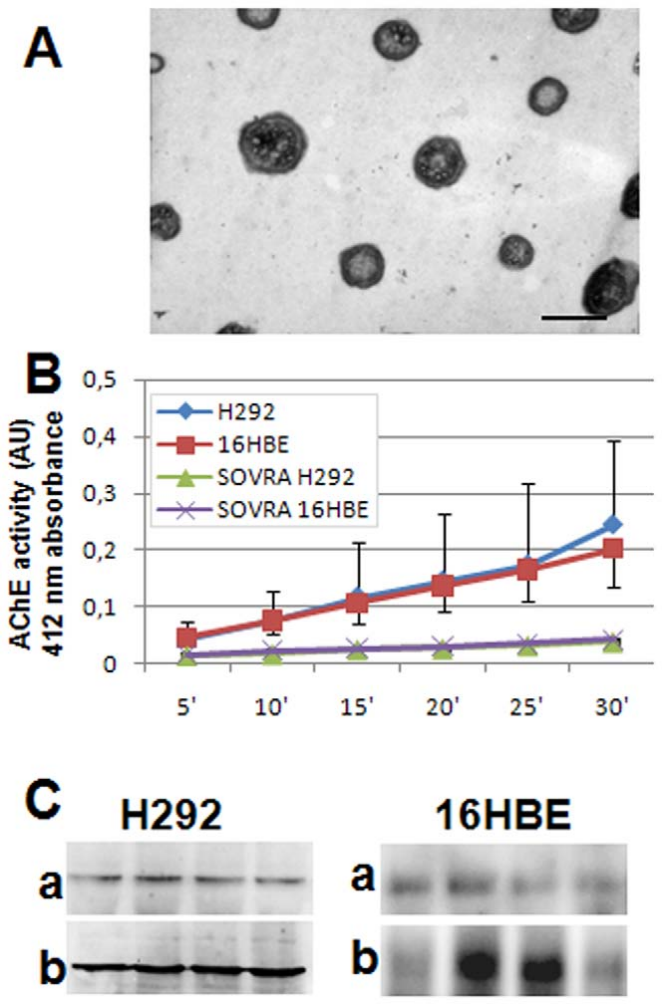
Characterization of exosomes: Morphology, acetylcholinesterase (AChE) activity, and Alix protein determinations. Vesicles compatible with typical exosomes constituted our exosomal preparations as demonstrated by TEM. An illustrative result for exosomes purified from H292 cells is shown in **A** (bar 50 nm). **B**) AChE activity in exosomal preparations from conditioned media and supernatants obtained after ultra-centrifugation (sovra) from tumor (H292) and non-tumor (16HBE) cells. Data are represented as mean ± SD of AChE activity (AU) measured at 412 nm every 5 min for 30 min in three independent experiments in duplicate. **C**) Demonstration of Alix protein by Western blotting in: (**a**) four exosomal preparations (one for each lane) from the conditioned media of the tumor cell line H292 and the non-tumor cell line 16HBE in four separate experiments; and (**b**) four whole-cell lysates from the same cell lines in four separate experiments.

Exosomes are vesicles of 30–90 nm in diameter that originate in the endosomal system [6]. Figure 2A shows that the exosomal preparations obtained presented the ultrastructural hallmarks typical of exosomes.

#### b) Measurement of exosome-associated acetylcholinesterase activity

AChE activity is typically detected in exosomes [7], [8]. Consequently, experiments aimed at establishing whether the exosomes purified from conditioned media showed AChE activity were carried out. Results are displayed in Figure 2B. The exosomes purified by ultra-centrifugation from culture media of tumor and non-tumor cell lines showed AChE activity that increased over the observation period of 30 min. In contrast, the supernatants obtained after ultra-centrifugation showed very low AChE activity in both cell lines throughout the observation period. These results were a further confirmation of the presence of exosomes in our extracellular samples.

#### c) Alix presence in extracellular exosomes and in the intracellular counterparts

Alix is a protein that is widely considered to be a valid marker of exosomes [9]; therefore, we decided to investigate its presence in the exosomes purified from the culture media. Figure 2C shows that this protein was indeed present in our exosomal preparations and, as expected, in the whole-cell lysates from tumor and non-tumor cell lines.

In conclusion, extracellular samples used in this study contained exosomes, as confirmed by the typical ultrastructural features observed with TEM, as well as by the presence of Hsp70 and Alix, and AChE activity; moreover, exosomes from tumor cell lines contained Hsp60 (Figure 1).

### Mechanisms Responsible for Hsp60 Release into the Extracellular Space

The data in the foregoing sections show that Hsp60 is found not only inside cells but also in the extracellular culture medium of tumor cell lines. We then proceeded to investigate whether the presence of Hsp60 outside the cells is simply due to cell death and membrane damage or to an active secretion mechanism in viable cells.

#### a) Selection and calibration of protein-secretion inhibitors

To begin dissecting the mechanisms involved in the secretion of Hsp60 in the cell models used in this study and to identify which protein-secretion machineries participate, we resorted to inhibitors of well characterized protein traffic pathways. This investigation was also fueled by our recent finding that Hsp60 is increased in H292 cells, suggesting that it is involved in tumor cell growth and cancer progression with a potential role of extracellular chaperone in these processes [10]. Therefore, the following experiments were carried out on H292 cells, using DMA and MBC (see Materials and Methods) to inhibit exosomes and lipid rafts, respectively.

In preparation for the inhibition tests, we measured (by MTT and AnnexinV assays, see Materials and Methods) the effects on viability/apoptosis of various doses and exposure times of DMA and MBC in H292 cells (data not shown). Based on the results of these preparatory experiments, non cytotoxic concentrations of the inhibitors were subsequently used. MTT assay results indicated that treatment of cells with DMA (5 nM) and MBC (1 mM) for one hour was not cytotoxic (DMA: 94.95+/−13.96% vs. untreated control; MBC: 106.06+/−10.25% vs. untreated control; p = 0.39 and p = 0.17, respectively). AnnexinV assay confirmed these results, since treatment with the inhibitors for one hour did not significantly change viability levels (DMA: 91.33+/−4.59% and MBC: 90.79+/−3.45% vs. untreated control: 89.7+/−3.89%; p = 0.43 and p = 0.52, respectively) and did not increase the percentage of early apoptotic cells (DMA: 1.6+/−0.3% and MBC: 2.3+/−0.7% vs. untreated control: 2.2+/−0.5%; p = 0.59 and p = 0.79, respectively). In conclusion, treatment with the inhibitors did not affect cell viability (necrosis and/or apoptosis) at the doses used.

#### b) Hsp60 and Hsp70 secretion inhibition

**Figure 3:**
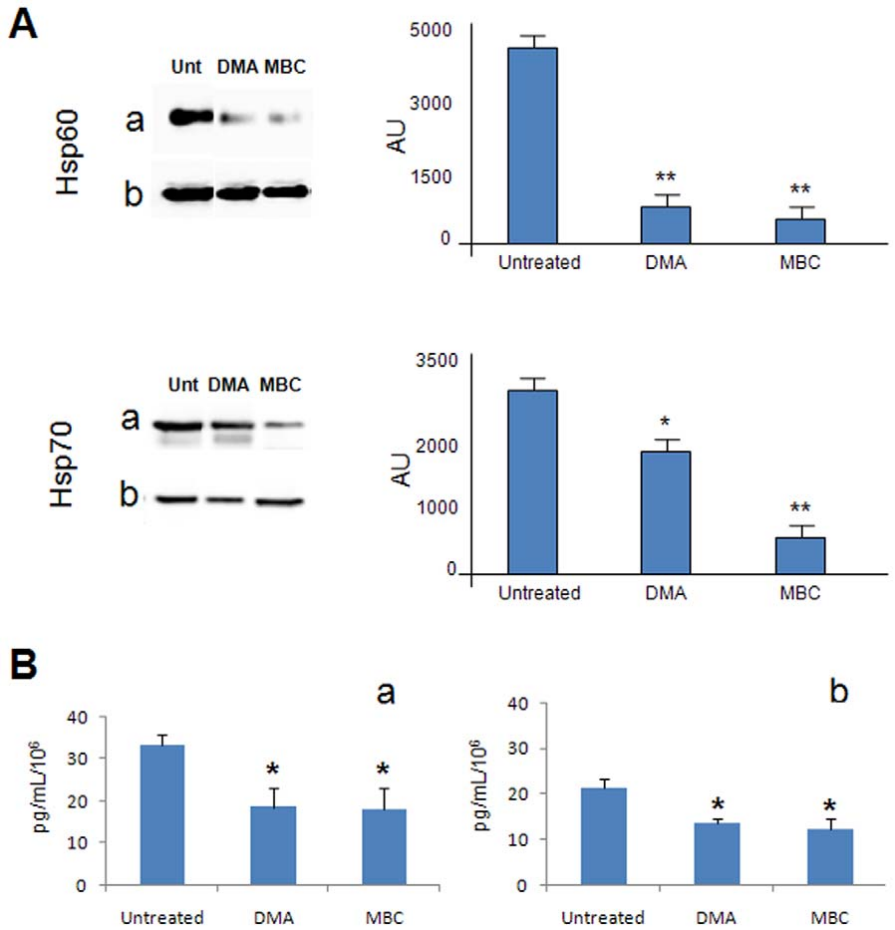
Effects of protein-secretion inhibitors on Hsp60 secretion by tumor cells. **A**) Hsp60 and Hsp70 detected by Western blotting in: (**a**) immunoprecipitates from conditioned media from untreated (Unt) and inhibitor-treated H292 tumor cells; and (**b**) whole-cell lysates from H292 cells. The inhibitors are listed on top of the respective lanes. Histograms to the right represent the levels of the Hsps in immunoprecipitates determined in three separate experiments as mean percentages +/− SD of arbitrary units (AU) obtained with NIH image J 1.40 analysis software. * and ** significantly different from untreated control, p<0.005 and p<0.001, respectively. The two inhibitors (listed below the bars) significantly decreased secretion of Hsp60 and Hsp70. Also, the data from whole-cell lysates show that the protein-secretion inhibitors had no detectable effect on Hsp levels inside the cells. **B**) Hsp60 levels secreted by the H292 tumor cells before and after exposure for 1 hour, followed by a 4 hours recovery period, to protein-secretion inhibitors measured by ELISA in: (**a**) conditioned media; and (**b**) exosomes. Histograms represent Hsp60 levels expressed as pg of protein normalized for mL normalised for 10^6^ cells. Data represent mean +/− SD of three different experiments in duplicate. * Significantly different from untreated control, p<0.005. The results, which are in agreement with those obtained by Western blotting, show that the inhibitors tested significantly reduced secretion of Hsp60 by the H292 tumor cells.

Immunoprecipitates were prepared with conditioned media from inhibitor-treated and untreated H292 cells, and tested as described under Material and Methods. A marked decrease in Hsp60 secretion was observed in cells treated with DMA (p<0.0001) or MBC (p<0.0001) (Figure 3A, top panel). These data suggest that exosomes and lipid rafts are actively involved in the release of Hsp60 into the culture medium. As expected, Hsp70 secretion was also considerably decreased after DMA (p<0.005) or MBC (p<0.0001) treatment (Figure 3A, bottom panel); these results are in accordance with those from other groups [11].

The findings reported in Figure 3A, indicate that the two Hsp appear in the extracellular space due to an active mechanism, and reinforce the hypothesis that lipid rafts may contribute to the exosomal machinery [12], [13]. Also of interest are the data from whole-cell lysates showing that protein-secretion inhibitors had no detectable effect on Hsp levels inside the cells (Fig. 3A, lanes b), suggesting that the inhibitors used did not affect protein synthesis and/or degradation. These data, together with what was observed earlier in terms of cell viability, suggest that extracellular Hsp60 basal levels and their decrease following treatment with inhibitors, could not have been influenced by dead cells or debris found in the extracellular milieu, but rather were due to intracellular active mechanisms and their alteration by the inhibitors.

### Quantification of Extracellular Hsp60 in Conditioned Media and Exosomes from H292 Tumor Cells before and after Exposure to Protein-Secretion Inhibitors

In order to confirm the results described in the previous sections by Western blotting, Hsp60 protein levels were quantified by ELISA in conditioned media and exosomes from H292 cells. Conditioned media and exosomes from H292 cells, untreated or treated with DMA or MBC, were collected and processed as described under Materials and Methods. Hsp60 levels in exosomes were 63.63% of the total amount of this protein found in the conditioned media (21.17 ng/mL/10^6^ cells vs. 33.2 ng/mL/10^6^ cells).

Treatment of H292 cells with the inhibitors (DMA or MBC) caused a significant reduction of Hsp60 levels in the conditioned media compared to untreated cells (p<0.001) (Figure 3Ba). Likewise, Hsp60 levels measured in exosomes from inhibitor-treated cells were significantly reduced compared to untreated controls, as shown in Figure 3Bb (DMA p<0.0001 and MBC p<0.001). In conclusion, the two different methods used, Western blotting and quantitative ELISA, showed agreement in the data obtained (Figures 3A and 3B), albeit with slightly different sensitivity. Both methods clearly showed that in our model the inhibitors decreased extracellular Hsp60 significantly, indicating that chaperonin release into the extracellular space was due to active mechanisms in which exosomes and lipid rafts were involved.

In order to demonstrate that DMA treatment specifically inhibited exosomal secretion, AChE activity was measured in the exosomal fractions after DMA treatment according to Savina et. al. [27]. Treatment of H292 cells with DMA, 5 nM for 1 hour, determined a significant reduction in AChE activity compared to the untreated control (0.095+/−0.023 vs. 0.245+/−0.148 OD, p<0.005).

To confirm the specificity of MBC treatment on lipid rafts depletion, cellular cholesterol concentration in H292 cells treated for 1 hour with MBC, 1 mM, was measured as described in the Materials and Methods section. MBC treatment caused a 66% reduction in cellular cholesterol content by comparison with the untreated control.

Our observations relate to three tumor cell lines that were selected for this study for specific reasons. H292 was used because its levels of intracellular Hsp60 are high, suggesting a tendency for extracellular release of this chaperone [10]. A549 was chosen because of its similarities with H292 (both cell lines are very similar cytomorphologically and, embryologically, the normal epithelium from which they derive is the same, i.e., lower airways from the primitive foregut), and we wanted to see whether what was observed using one cell line could be reproduced in another comparable one. Lastly, K562 was incorporated in our research because it is quite distinct from the other two cell lines mentioned above, since it does not represent a solid tumor, or epithelial cells, or cells from the respiratory tract. We wanted to test if the observations made with the other two cell lines could be repeated in a tumor cell line with different origin and phenotype. Since all cell lines released Hsp60, we hypothesize that our observations are likely to have general validity and apply to a variety of tumors. This hypothesis is also supported by other arguments, briefly discussed below.

### Considerations and Conclusions

The role of extracellular Hsp60 in healthy and diseased tissue is still unclear. Hsp60 has been found on the cell-membrane's outer surface in both normal [14] and tumor cells [15], [16]. It has been implicated in transmembrane transport and signaling [14], [17], activation and maturation of dendritic cells with generation of antitumor T-cell responses [15], [18], as well as in triggering apoptosis [19]. Hsp60 may also be secreted from cells into the interstitial fluid and thereby reach the bloodstream [20], where its levels may be under genetic control [21]. In view of these data in the literature, it is likely that the Hsp60 secretion reported in this work is not an isolated phenomenon, a peculiarity of the cell lines tested. On the contrary, all published information suggests that Hsp60 secretion must occur often *in vivo*, in a variety of tumors and situations, because this extracellular chaperone seems to be a part of widespread intercellular communication events with profound biologic effects. However, it remains to be determined where circulating Hsp60 from tumor cells goes and what does it do at its destination (or various destinations).

In regard to the secretion mechanisms for Hsp60 from tumor cells, our studies contribute new data to an area still poorly characterized. In the first place, we have shown that the presence of Hsp60 in the extracellular media is not due to cell death and destruction, but to an active secretion mechanism. Concerning the latter mechanism, there is information indicating that Hsp70 is secreted by normal and tumor cells via exosomes [4], [22], but the situation is less clear for Hsp60. Few years ago, it was reported that exosomes obtained from a human B-lymphoblastoid cell line, either before or after stress, did not contain Hsp60 [23]. In contrast, other researchers have reported that exosomes derived from a heat-shocked mouse B-cell lymphoma contained more Hsp60 than the unstressed cellular counterparts [24]. More recently, it was reported that Hsp60 is secreted by normal adult rat cardiomyocytes through the exosomal pathway [20]. The part played by lipid rafts in Hsp60 secretion by tumor cells is not completely understood either. Lipid rafts are believed to be involved in the process of exosome formation [12], [13] and, in this scenario, it is pertinent to mention that an association between Hsp60 and lipid rafts of stressed endothelial cells has been postulated [25]. Thus, it would be safe to assume that the results obtained with a lipid raft inhibitor corroborate the data obtained with an exosome inhibitor, since it is likely that both inhibitors hit two different stages of the exosome pathway.

It is clear at the present time that Hsp60 in the cell membrane is rare in normal cells but frequent in tumor cells [1], [2]. The finding that Hsp60 secretion is inhibited not only by DMA but also by MBC, which is a lipid rafts inhibitor, supports the notion that this chaperonin occurs in the cell membrane of tumor cells, although it is not understood how Hsp60 reaches the cell membrane. We hypothesize that the presence of Hsp60 in the cell membrane is one of the key requirements for its secretion via the “lipid rafts-exosomes” pathway. Also, it remains to be established why Hsp60 is released via exosomes by the tumor (i.e., H292) but is not by the non-tumor (i.e., 16HBE) cells despite the fact that both are capable of producing Hsp60 and exosomes.

## Materials and Methods

### Cell Cultures and Reagents

NCI-H292 (human mucoepidermoid bronchial carcinoma), A549 (human lung adenocarcinoma) and K562 (human erythroleukemia) cell lines, referred to as “tumor cells” or “tumor cell lines,” were obtained from the American Type Culture Collection and maintained in RPMI 1640 with 10% heat-inactivated fetal calf serum (FCS). We also used the SV40 large T antigen–transformed 16HBE cell line, referred to as “non-tumor cells” or “non-tumor cell line,” a human bronchial epithelial cell line that retains the differentiated morphology and function of normal human airway epithelium [26]. The 16HBE cells were cultured in Dulbecco-modified Eagle's medium with 10% FCS and supplemented with 2 mM glutamine, 50 U/ml penicillin, and 50 mg/streptomycin. All cell lines, except K562, were grown as monolayers attached to the culture vessel and cultured at 37°C, 5% CO_2_ in a humidified incubator. Passage number of cells used in this study ranged from 12 to 35. Unless otherwise stated, cell culture reagents were purchased from GIBCO BRL LIFE Technologies (Invitrogen, Italy). Prior to all the experiments, confluent cell monolayers were incubated in serum–free medium (to avoid possible contaminations by bovine exosomes from the FCS) for the indicated times.

### Protein-Secretion Inhibitors

5,5-(N-N-Dimetyl)-amiloride hydrochloride (DMA), an exosomal inhibitor [27], and methyl-β-cyclodextrin (MBC), a lipid-raft pathway inhibitor - since it depletes cellular cholesterol and disrupts lipid rafts [11] - were purchased from Sigma (Italy).

### Antibodies

Anti-Hsp60 (clone LK1) monoclonal antibody was purchased from Sigma and used diluted 1∶1,000; anti-Hsp70 (clone W27) monoclonal antibody was purchased from Santa Cruz Biotechnologies (CA, USA) and used diluted 1∶1,000; Anti-Alix monoclonal antibody was purchased from Pharmingen (BD Biosciences, CA, USA) and used diluted 1∶500. Control mouse immunoglobulins with isotypes matching those of the anti-Hsp antibodies were purchased from Sigma, and used before immunoprecipitation with the anti-Hsp antibodies to remove proteins from the media that bind immunoglobulins non-specifically [28]. Horseradish peroxidase (HRP)-conjugated sheep anti-mouse antibody and Protein-A Sepharose were purchased from Amersham Biosciences (Ge Healthcare, Italy).

### Preparation of Extracellular Fractions from Cell Cultures Exposed to Protein-Secretion Inhibitors and Untreated Controls

#### a) Conditioned medium

Eighty ml of conditioned medium from 70–80×10^6^ cells was collected after 24 hrs of culture in serum-free medium, and centrifuged (800×g for 10 min) to eliminate cells and debris that might have been present. Thirty ml of this medium was then dialyzed in a buffer of 50 mM NaCl plus 0.05% Nonidet at 4°C O/N, subsequently lyophilized and stored at 4°C, and resuspended just before use into a solution containing RIPA buffer (25 mM Hepes, 300 mM NaCl, 150 µM MgCl, 0.2 mM EDTA, 1% Triton X, 0.25% deoxycholate, 0.05% SDS and protease inhibitors cocktail; Roche, Italy). Five hundred µg of proteins in this resuspended solution (representing the conditioned medium *in toto*, referred to as “conditioned medium”) was used for immunoprecipitation and, in turn, the immunoprecipitate (referred to as “immunoprecipitate” as described later, under **Immunoprecipitation**) was used for Western blotting to quantify Hsp. Fifty ml of the remaining resuspended solution (conditioned medium) was not immunoprecipitated, but used for exosome isolation as described below.

#### b) Exosomes purification

Exosomes were isolated according to a previously published method [27]. Briefly, 50 ml of cell- and debris-free conditioned medium (see above, **(a) Conditioned medium**) was collected on ice and centrifuged at 13,000×g for 20 min to bring down and eliminate small cellular debris and mitochondrial contaminants that might have been present. The supernatant was collected and exosomes were separated from it by centrifugation at 110,000×g for 2 hrs in a Beckman 60 Ti rotor; the pellet (exosomal fraction) was saved and washed once in phosphate-buffered saline (PBS), resuspended in 100 µl of PBS containing protease inhibitors, and stored at −80°C until use; supernatants after ultra-centrifugation were stored at −80°C and used for AChE activity determination.

#### c) Exosomes validation by Transmission Electron Microscopy (TEM)

Exosome pellets purified from all cell lines studied were examined by TEM to ascertain the presence of exosomal vesicles. Pellets were resuspended in residual fluid from a PBS wash, followed by addition of 100 µl ice-cold, freshly made fixative (2.5% glutaraldeyde in PBS) to preserve vesicle structure and morphology. Preparations were mounted on formvar nickel 300-mesh grids by layering grids over 10-ml drops of exosome preparations for 10 min at room temperature. Grid-mounted preparations were stained with uranyl acetate and lead citrate, and subsequently examined with a Jeol (JEM 1220) TEM at 120 kV.

#### d) Exosomes validation by acetylcholine esterase assay and protein Alix determination

The presence of exosomes was confirmed by measuring the activity of acetylcholineesterase (AChE), an enzyme that is considered a marker for these vesicles [7] and by measuring the protein Alix, which is also considered a marker of exosomes [9]. Briefly, for the AChE assay, 15 µl of the exosomal preparation was suspended in 100 µl of PBS and incubated with acetylthiocholine (1.25 mM) and 5,5′-dithio-bis-(2-nitrobenzoic acid) (0.1 mM) in a final volume of 1 ml. The incubation was carried out in cuvettes at 37°C and the change in absorbance at 412 nm was monitored every 5 minutes for 30 minutes. For Alix detection, we used Western blotting with a specific antibody (see section ***Antibodies***). Acetylthiocholine iodide and 5,5′-dithio-bis-(2-nitrobenzoic acid) were purchased from Sigma.

### Intracellular Proteins: Whole-Cell Lysate Preparations from Cell Cultures Treated with Protein-Secretion Inhibitors and Untreated Controls

Treated and untreated cells were lysed into ice-cold lysis solution containing RIPA buffer, as previously described [28]. Lysates were then spun at 16,000×g for 30 min at 4°C, the supernatant was recovered, its protein concentration determined, and then stored at −80°C until use. This preparation is referred to as “whole-cell lysate.”

### Protein Quantification

Proteins were quantified in conditioned media and corresponding immunoprecipitates - see following section -, exosomal preparations, as well as in whole-cell lysates from inhibitor-treated and untreated control cultures with the Quant-iT™ protein assay kit (Invitrogen Molecular Probes, Italy), using the Qubit fluorometer according to the manufacturer's instructions (the kit is accurate for protein concentrations ranging from 12.5 µg/ml to 5 mg/ml).

### Immunoprecipitation

To assess the presence and quantify Hsp60 in the “conditioned medium” (namely, the resuspended lyophilized powder described above under **Preparation of extracellular fractions. a) Conditioned medium**), 500 µg protein was incubated with 3 µg of anti-Hsp60 antibody at 24°C for 2 h, followed by incubation with 20 µl protein-A Sepharose at 4°C for 12 h. Subsequently, the incubation mixture was centrifuged in a microcentrifuge at 14,000×g for 30 sec at 4°C, the pellet was collected and resuspended in lysis buffer and centrifuged again: this procedure was repeated three times. The last pellet was solubilized by boiling into 2x sample buffer (2% SDS, 10% glycerol, 100 mM DTT, 60 mM Tris-HCl [pH 6.8] and 0.001% bromophenol blue), and used for SDS-PAGE as described under Western blotting. The final immunoprecipitate preparation is referred to as “conditioned medium Hsp60-immunoprecipitate.” The same protocol, using anti-Hsp70 instead of the anti-Hsp60 antibody, was applied to assess Hsp70; the final preparation obtained is referred to as “conditioned medium Hsp70-immunoprecipitate.” All immunoprecipitates were tested by Western blotting.

### Western Blotting

Western blotting analyses of immunoprecipitates, exosomes, and whole-cell lysates were performed as previously described [28]. Briefly, 40 µg of proteins from immunoprecipitates or whole-cell lysates, or 25 µl of exosomes (protein concentrations ranging from 0.45 to 1 µg/ml) was added to 4x Laemmli buffer and heated for 5 min at 95°C. Proteins were resolved by 12% SDS-PAGE along with a molecular weight marker (Bio-RAD laboratories, Italy). Proteins were then transferred to nitrocellulose membranes. After transfer, all membranes were stained with Poinceau S to verify the quality of transfer and loading similarity. Before applying antibodies, the membranes were blocked with 5% fat milk, and probed for 12 hours with the specific antibody, followed by incubation with horseradish peroxidase-conjugated second antibody. Blots were detected using the Supersignal West Femto, according to the manufacturer's instructions (Pierce, Italy), and chemiluminescent signals were recorded with the ChemiDoc XRS imager (Bio-RAD Laboratories). Densitometric analysis of blots was performed using the NIH Image J 1.40 analysis program (National Institutes of Health, Bethesda, MD, USA).

### Treatment with Inhibitors

Cells were seeded in serum free medium under the same conditions described previously and treated with 5 nM DMA or 1 mM MBC for 1 hour, followed by 4 hours recovery period. Extracellular medium was processed as described before and was used for immunoprecipitation and for purification of exosomes.

### MTT Assay to Assess Cell Viability

MTT [3-(4,5-dimethylthiazol-2-yl)-2,5-diphenyltetrazolium bromide] was obtained from Sigma (Italy), and the assay was performed as described [29]. Briefly, 5×10^3^ H292 cells were plated in 200 µl of complete (with FCS) medium per well in 96-well plates. After 24 hrs, complete medium was replaced with FCS-free medium, and the protein secretion inhibitor DMA (at 30, 15, 7, and 5 nM), or MBC (at 5, and 1 mM) was added for one hour. The medium containing the inhibitor was replaced, the cells were left to recover in inhibitor-free medium for 4 hours and, at the end of this incubation period, MTT was dissolved in fresh medium and added to the cell cultures at a final concentration of 0.5 mg/ml. Following a 4-hour incubation period, cells were solubilized in 200 µl DMSO/well and optical density (OD) was measured with a plate reader (*Titertek* Multiskan MCC/*340*, Flow Laboratories, Switzerland) at 570 nm (630 nm as reference). Cell viability was expressed as the percentage of the OD value of inhibitor-treated cells compared with untreated controls, according to the following equation: Viability = (OD SAMPLE/OD CONTROL)×100. Each experiment was carried out in duplicate and a total of three experiments were performed for each inhibitor.

### Early Apoptosis Determination

Fifty thousand H292 cells/well were plated into 24-well plates and grown until 80% confluence. Growth medium was replaced with FCS-free medium for 24 hours and cells were then treated with protein-secretion inhibitor DMA (5 nM) or MBC (1 mM) for one hour, followed by a 4-hour recovery period in inhibitor-free medium. These times and doses were chosen according to the MTT assay results as described in the Results section. H292 cells were then harvested for early apoptosis analysis according to a previously published technique [30], in which binding of Annexin V (AxV) is used to detect phosphatidylserine that is externalized on the outer leaflet of the plasma membrane of apoptotic cells. AxV-FITC (1 µg/ml) and propidium iodide (PI, 2.5 µg/ml) were added to tubes containing 1×10^5^ cells/100 µl binding buffer. Cells were incubated in the dark for 15 min, and then analyzed using a FACScan flow cytometer (Becton Dickinson, Oxford, UK). Control tubes lacking either AxV-FITC or PI, or both, were included to complete the controls. Analysis of dot-plots of fluorescence detector (FL) 1 (AxV-FITC) versus FL2 (PI) was performed using Win MDI 2.8 (Flow cytometry software, University of Massachusetts, MA, USA). The degree of early apoptosis was expressed as the number of AxV^+^/PI- cells shown as the percentage of total cells. Each experiment was carried out in duplicate and a total of three experiments were performed.

### Cholesterol Depletion

To deplete cellular cholesterol, cells were treated with MBC as describe above. The cell pellets were collected, lysed, and the intracellular cholesterol concentrations were determined using the pertinent Roche Kit according to the manufacturer's instructions (Roche, Milan, Italy).

### ELISA

Quantitative comparisons of Hsp60 levels in conditioned media and exosomes by ELISA were performed using a commercial Hsp60 EIA Kit from Stressgen Assay Designs Inc (Ann Arbor, MI, USA). The results obtained with this assay were normalized for cell number and expressed as pg\mL\10^6^ cells.

### Statistical Analysis

Data are presented as the mean ± SD of triplicate determinations. Comparisons between groups were performed using the unpaired samples Student's *t* test. A *p* value≤0.05 was considered statistically significant.
